# Opioid-Use, COVID-19 Infection, and Their Neurological Implications

**DOI:** 10.3389/fneur.2022.884216

**Published:** 2022-05-23

**Authors:** Richa Jalodia, Danielle Antoine, Regina Gonzalez Braniff, Rajib Kumar Dutta, Sundaram Ramakrishnan, Sabita Roy

**Affiliations:** Department of Surgery, University of Miami Miller School of Medicine, Miami, FL, United States

**Keywords:** ACE2, COVID-19, CNS injury, opioid use and abuse, microbiome and dysbiosis

## Abstract

Severe acute respiratory syndrome coronavirus 2 (SARS-CoV-2) is an imminent threat to human health and public safety. ACE2 and transmembrane serine protease 2 proteins on host cells provide the viral entry point to SARS-CoV-2. Although SARS-CoV-2 mainly infects the respiratory system, there have been reports of viral neurotropism and central nervous system injury as indicated by plasma biomarkers, including neurofilament light chain protein and glial fibrillary acidic protein. Even with a small proportion of infections leading to neurological manifestation, the overall number remains high. Common neurological manifestations of SARS-CoV-2 infection include anosmia, ageusia, encephalopathy, and stroke, which are not restricted to only the most severe infection cases. Opioids and opioid antagonists bind to the ACE2 receptor and thereby have been hypothesized to have therapeutic potential in treating COVID-19. However, in the case of other neurotropic viral infections such as human immunodeficiency virus (HIV), opioid use has been established to exacerbate HIV-mediated central nervous system pathogenesis. An analysis of electronic health record data from more than 73 million patients shows that people with Substance Use Disorders are at higher risk of contracting COVID-19 and suffer worse consequences then non-users. Our *in-vivo* and *in-vitro* unpublished studies show that morphine treatment causes increased expression of ACE2 in murine lung and brain tissue as early as 24 h post treatment. At the same time, we also observed morphine and lipopolysaccharides treatment lead to a synergistic increase in ACE2 expression in the microglial cell line, SIM-A9. This data suggests that opioid treatment may potentially increase neurotropism of SARS-CoV-2 infection. We have previously shown that opioids induce gut microbial dysbiosis. Similarly, gut microbiome alterations have been reported with SARS-CoV-2 infection and may play a role in predicting COVID-19 disease severity. However, there are no studies thus far linking opioid-mediated dysbiosis with the severity of neuron-specific COVID-19 infection.

## Introduction

Severe acute respiratory syndrome coronavirus 2 (SARS-CoV-2) which emerged in China in late 2019 is the causative agent for the global coronavirus disease 19 (COVID-19) pandemic. COVID-19 severity, ranging from asymptomatic to lethal, is an imminent threat to human health and public safety (1). SARS-CoV-2 is a novel coronavirus belonging to the Coronaviridae family and is a single stranded ribonucleic acid (RNA) virus that shares a close resemblance to the bat-derived coronavirus strains (2). In the past two decades, three highly pathogenic zoonotic coronaviruses, SARS-CoV, 2002, Middle East respiratory syndrome coronavirus (MERS, 2012) and SARS-CoV-2, 2019 have emerged as major public health concerns (3).

On an average, it takes 5-6 days for COVID-19 symptoms to appear, with fever, cough, and tiredness being the most common symptoms, and sore throat, headache, and diarrhea being less common. A subgroup of COVID-19 survivors (~10%) experience long-COVID or post-COVID syndrome with symptoms persisting beyond the acute phase of the disease, including fatigue, myalgia, reduced physical activity, dyspnea, long-term anosmia, and ageusia (4, 5). Post-COVID syndrome also includes sequelae affecting the mental health of the survivors (6). Symptoms include depression, anxiety, mood swings, and cognitive symptoms impacting memory and attention deficits (7). Prolonged cognitive impairment is observed in both severely ill COVID-19 patients and those with relatively mild symptoms (8) and referred to as “COVID long haulers.” This long-term effect of disease lasting for years was also observed in previous coronavirus outbreaks with SARS-CoV and MERS (9).

The presence of SARS-CoV-2 viral RNA has also been detected in a few cases in the cerebrospinal fluid (CSF) of patients as well as animal models (10, 11). However, even in absence of detectable viral RNA in brain, evidence of encephalitis, neuroinflammation and neuronal damage is reported (12). Central nervous system (CNS) histopathology of SARS-CoV-2 infection includes hypoxic/ischemic encephalopathy, microglial activation, T cell infiltration, acute myelitis, encephalitis, demyelinating disorder with report of lesion in some cases (13, 14). Hypoxia/ ischemic encephalopathy related clinical symptoms such as headache, dysphoria, delirium, confusion loss of consciousness are the most common correlates in COVID-19 patients (15).

Pre-existing comorbidities such as hypertension and diabetes mellitus are mostly observed in more than one third of post-COVID syndrome patients (16). Factors such as viral load, tissue angiotensin-converting enzyme 2 (ACE2) density, vascular permeability, and cytokine activation cascade can have a possible role in determining the incapacitating clinical picture in patients suffering from post-COVID syndrome (17). Accumulating evidence also highlight the role of microbiota in determining COVID-19 disease severity and recovery (18–23). Opioid use disorder (OUD) has been associated with factor such as respiratory depression, immune modulation, microbial dysbiosis, and bacterial translocation, which can influence COVID-19 disease progression and severity. In this review, we will explore the role of opioid use in the rate and severity of COVID-19 infection.

## Review Methods

We performed a comprehensive literature review of COVID-19, OUD, and microbiome using PubMed, Google scholar, and SCOPUS databases using the keywords: “microbiome,” “SARS-CoV-2,” “Coronavirus,” “Opioids,” and “Opioid use disorder.” References listed in review articles and original articles were scanned for relevant studies.

**Figure 1:**
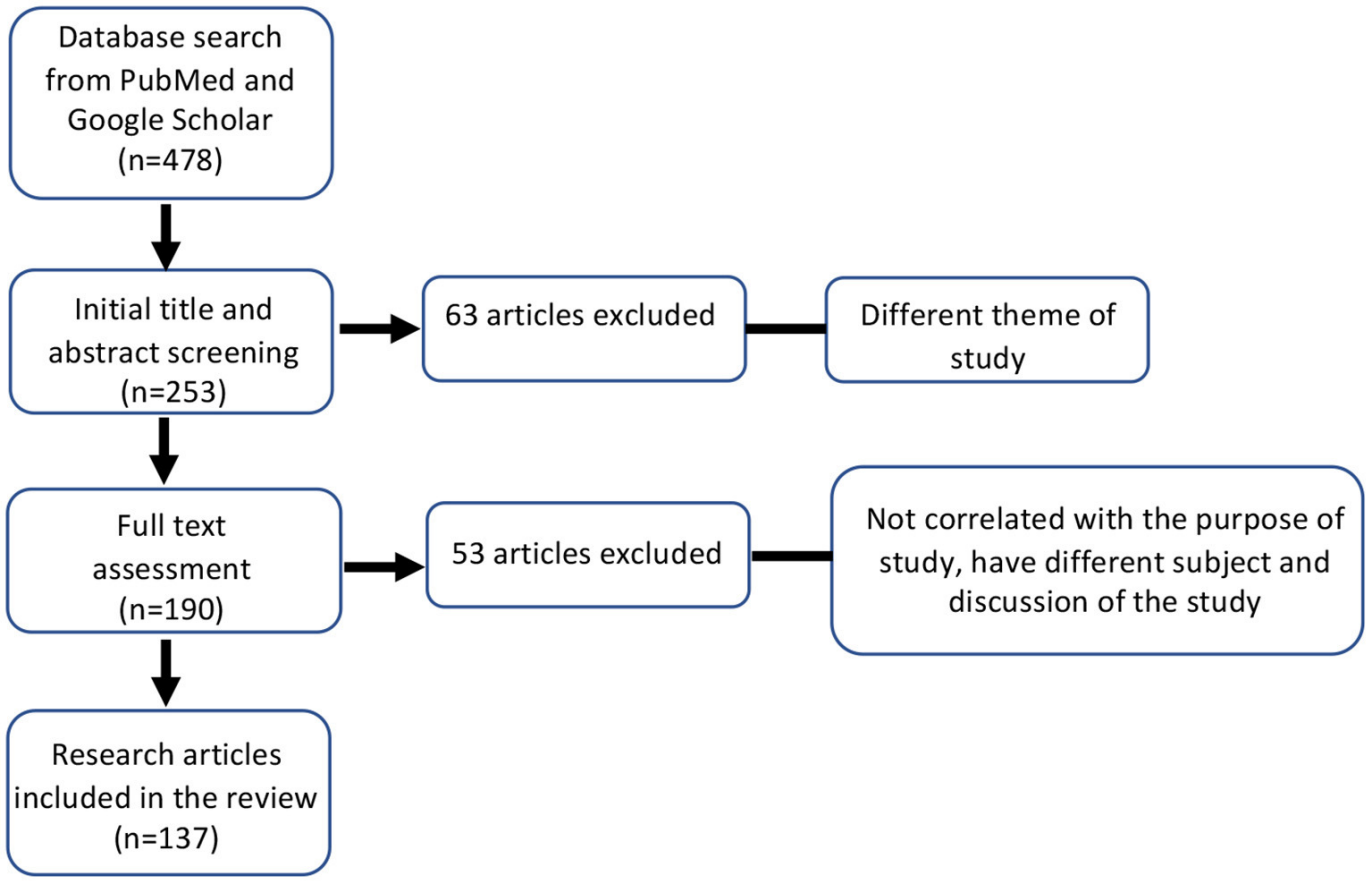
Flow-chart of the literature search and inclusion/exclusion criteria used in this study. ***P ≤ 0.001.

In total, 478 articles were retrieved from all databases. Initial screening based on title of the study excluded 225 articles. Based on the abstract and title, 63 articles were found to be irrelevant and were excluded from the study. We conducted a full-text assessment of 190 review and research articles. A secondary exclusion based on articles having a different subject or discussion and those that were not in line with the purpose of the review. We included 137 articles investigating COVID-19, Opioids, and the microbiome (Figure 1).

## Clinical And Epidemiological Features of SARS-CoV-2 Infection

COVID-19 disease can have diverse clinical manifestations in individuals based on their age, gender, ethnicity, and the presence of underlying health conditions such as cardiovascular disease, diabetes, cancer, and kidney damage (24–26). Infection symptoms for COVID-19 range from mild symptoms in 80% of patients to severe illness in 14% of patients and critical illness in 5% of symptomatic patients. Typical symptoms of COVID-19 infection as listed by CDC include fever (58.66%), cough (54.52%), dyspnea (30.82%), fatigue (28.16%), malaise (29.75%), sputum secretion (25.33%) chills (13.49%), muscle pain (16.9%), sore throat (14.41%), and diarrhea (9.59%) (27, 28). New loss of taste or smell has also been added to the list of COVID-19 symptoms. In a temporal study, a subgroup of COVID-19 patients showed persistent radiographic and physiological changes in the lung over a period of 12 months, which were reflected in their pulmonary function, suggesting a long-term effect of the infection (29).

Most symptomatic patients start showing symptoms of SARS-CoV-2 from 2-14 days of infection (27). Among critically ill COVID-19 patients, the average time for developing dyspnea was 5-8 days, acute respiratory distress syndrome (ARDS) was 8–12 days, and intensive care unit (ICU) admission was 9.5–12 days from the onset of illness (30, 31). Based on current data, the case fatality rate for COVID-19 infection seems to be lower than that of MERS (34.4%) and SARS (9.5%), but higher than influenza (0.1%) (32). Age is the strongest risk factor for severe COVID-19 illness, with a case fatality ratio of 8% in people above 80 years (32–34). In addition, 29–91% of critically ill COVID-19 patients admitted to ICU need invasive mechanical ventilation and are at higher mortality risk (35–37). The average length of hospitalization among COVID-19 survivors was 10-13 days and those who spend more than 30 days in hospitals have an increased risk of death. With long-term ICU stay, the bacterial and fungal superinfections rate also increases in COVID-19 patients (38, 39).

Critically ill COVID-19 patients have been recorded to have high cytokine concentrations in plasma, referred to as a “cytokine storm,” triggering systemic inflammation and leading to multi-organ failure and death (40). Cytokine storm also referred to as cytokine release syndrome (CRS), is a systemic inflammatory response by monocytes and macrophages activated by infectious agents. These activated monocytes and macrophages trigger leukocyte recruitment and production of inflammatory mediators, which play a major role in the severity of SARS-CoV-2 infections and lung injury (41). IL-6 is the most frequently reported pro-inflammatory cytokine found to be elevated in the serum of severe SARS-CoV-2 patients (42, 43). An elevated level of several other pro-inflammatory cytokines, including tumor necrosis factor-α (TNFα), IL-8, IL-1β, IL-10, IL-2, IL-4, soluble IL-2 receptor (sIL-2R), and interferon-γ (IFNγ) were also observed in severe COVID-19 patients. However, the levels of pro-inflammatory cytokines, including IL-6 during SARS-CoV-2 cytokine storm remain profoundly lower than ARDS, sepsis, chimeric antigen receptor (CAR) T-cell induced CRS, and influenza (44–46). In addition, serum levels of several other biomarkers such as D-dimer, C-reactive protein, and ferritin were also elevated in COVID-19 patients (40).

## Neurological Aspect of SARS-CoV-2 Infection

As the number of patients with COVID-19 increased, several neurological manifestations have been reported. The SARS-CoV-2 virus seems to damage the CNS through direct entry into the CNS (10). The spike (S) protein of CoV binds to the ACE2 receptor on the host cell surface to facilitate viral entry. Various cells in the CNS express ACE2 along with the other receptors needed for viral entry. These cells include the endothelial cells (47), which are part of the blood-brain barrier (BBB), neuronal cells, and the neuroglia, including the microglia and astrocytes (48, 49). Such co-expression in the neurovascular unit facilitates SARS-CoV-2 infection in the CNS. In addition to direct entry, the SARS-CoV-2 virus causes a systemic inflammation with a “cytokine storm” that reaches the CNS and results in damage (12, 50). The predominant neurological syndromes of COVID-19 include anosmia, ageusia, encephalopathy, and stroke (51). The most common neurological symptom of SARS-CoV-2 infection is anosmia, and, in most cases, it occurs even in the absence of other symptoms. The underlying mechanisms of this symptom include the expression of ACE2 in the supporting and non-neuronal cells in the olfactory system. In both human and mouse samples, ACE2 was significantly expressed in the supporting cells of the olfactory system but not in the sensory neurons (52). Thus, such expression of these genes in the non-neuronal cells of the olfactory system seems to be responsible for the anosmia and the other issues in odor perception experienced by COVID-19 patients. The other important neurological symptom of SARS-CoV-2 infection is encephalopathy, which can alter higher cognitive function. The term encephalopathy is used to describe “a rapidly developing (in < 4 weeks) pathobiological brain process which is expressed clinically as either subsyndromal delirium, delirium or coma and may have additional features, such as seizures or extrapyramidal signs” (53). In a study looking at encephalopathy and neurological symptoms of COVID-19 in ICU patients, 118 of 140 patients (84.3%) developed delirium with a combination of acute attention, awareness, and cognition disturbances, and 88 patients (69.3%) presented an unexpected state of agitation despite high infusion rates of sedative treatments and neuroleptics. Only 22 (15.7%) patients had normal neurological examination (54). Such neurological symptoms may be due to a systemic inflammatory reaction to SARS-CoV-2, as the previous study found inflammatory disturbances in two-third of the patients when analyzing their CSF.

Additionally, SARS-CoV-2 viral elements were found within endothelial cells, along with an accumulation of inflammatory cells and evidence of endothelial and inflammatory cell death (55). Consequently, the resulting endotheliitis can cause disruptions in the blood-brain barrier and may explain the inflammatory component of the brain in response to SARS-CoV-2 infection. Another neurological syndrome associated with COVID-19, which may not be as prevalent but occurs in many patients, is an acute cerebrovascular disease (CVD). A recent paper reported that out of 108,571 patients with COVID-19, acute CVD occurred in 1.4% of the patients. The most common manifestation was acute ischemic stroke (87.4%). Patients with COVID-19 developing acute cerebrovascular diseases were older and more likely to have hypertension, diabetes mellitus, coronary artery disease, and severe infection (56). Interestingly, COVID-19 does not worsen psychiatric or neurological impairments in some neuropsychiatric disorders such as Wilson's disease (57). However, research is still limited on the effect of COVID-19 on other neuropsychiatric diseases.

## Opioid Use, Respiratory Depression, And The SARS-CoV-2 Epidemic

Opioids are commonly used in the ICU and in mechanically ventilated patients for sedation and analgesia (58). Invasive mechanical ventilation involves using sedatives to reduce discomfort and distress, and prevent the patient from unintentionally extubating (58). Due to concerns about the adverse effects of benzodiazepines and Propofol, such as delirium and hypotension, updated guidelines have recommended reducing their use (59). As a result, opioids are almost always used as the routine sedative and analgesic agent for mechanically ventilated patients (59). According to observational studies, 85% of mechanically ventilated patients received opioids as part of their treatment (59). However, the use of opioids in these circumstances can have adverse effects and extend a patient's time in the ICU and on mechanical ventilation (58).

According to a recent study published in NEJM, 100% of COVID-19 patients admitted due to ARDS were given opioids for pain management along with Midazolam (86% of patients) or Propofol (47% of patients) (60). Opioid treatment is routinely used as palliative care in COVID-19 patients, and according to a recent study, patients with post-COVID have a high rate of opioid use post pain management. According to a recent study, for every 1000 post-COVID patients at US Veteran Health Administration (VHA), 9 more patients received opioid prescriptions than normally would. They also received other prescription medications for benzodiazepines, such as Xanax for anxiety (61). The global COVID-19 pandemic is exacerbating the opioid epidemic in the USA. According to a recent retrospective case-controlled study, OUD increased the risk for COVID-19 infection [adjusted odds ratio = 10.244 (9.10–11.52), *P* < 10^−30^] and also resulted in worse outcomes in these patients (death: 9.6%, hospitalization: 41.0%, *P* < 0.05) (62). People with OUD with or without pre-existing medical conditions are more vulnerable to severe COVID-19 infection due to poor health. Additionally, individuals with OUD are also at higher risk of getting infected and transmitting SARS-CoV-2, because drug procurement and drug use practices often require social contact.

Opioid use and abuse have been established to cause several comorbidities, increasing the risk of developing severe illness among COVID-19 patients. Respiratory depression is a serious adverse effect of opioid use, often leading to respiratory arrest and death (63). Opioid-induced respiratory depression is a consequence of the activation of μ opioid receptors (MOR) expressed on neurons in the brainstem (63). Morphine can cause hypoventilation even at low doses, significantly lowering respiratory rate and airflow and reducing ventilatory response to carbon dioxide (64). Therefore, together with opioid use, the impact of COVID-19 infection on the lungs may lead to worse outcomes (65).

Acute respiratory distress syndrome results from a disrupted pulmonary barrier caused by underlying inflammation and can be triggered by opioid abuse (66). About 10% of patients in the ICU develop ARDS, often resulting in death. A study using MOR agonist, endorphin-1, showed a protective effect on lung tissue after LPS induced pulmonary injury, characteristic of ARDS (66). Endorphin-1 is an endogenous opioid agonist. Endogenous opioids have been shown to have anti-inflammatory properties and promote immune function, as opposed to exogenous opioids (67, 68). These findings suggest that exogenous opioids may have the opposite effect, exacerbating ARDS. Interestingly, opioids are often used to treat patients with ARDS. A study showed that >80% of patients with ARDS were given opioids, and this treatment was associated with prolonged mechanical ventilation and worse outcomes (69). Clinical data needs to be collected and analyzed to understand the impact of opioid use on respiratory function in the context of COVID-19.

## Gut Microbial Alterations in OUD and SARS-CoV-2 Infection

Several recent studies have found that SARS-CoV-2 infection is associated with altered gut microbiota composition, serving as a prognosis tool for COVID-19 disease severity (18–23). Bacteroidetes phylum was most abundant in patients with COVID-19, and Actinobacteria was found to be depleted (22). Gut microbiota composition between COVID-19 ICU patients and COVID-19 infectious disease ward patients also varied with a significant increase in bacterial phylum *Staphylococcaceae, Microbacteriaceae, Micrococcaceae, Pseudonocardiaceae, Erysipelotrichales* (19). Studies also reported the presence of SARS-CoV-2 virion in fecal samples of COVID-19 patients even after having a negative result in respiratory samples suggesting a possible oral-fecal contamination route (70, 71). Metagenomic analysis of gut microbial composition in COVID-19 patients revealed a significant reduction in overall bacterial diversity with a marked increase in opportunistic pathogenic species and a decrease in commensal bacterial species (18, 72) (Table 1). Furthermore, recently published studies also show secondary bacterial infection and bacterial translocation among 12–14% of COVID-19 patients (74, 75). Decreased microbial richness and microbial dysbiosis observed in COVID-19 patients may persist for a long duration even after recovery and respiratory clearance of the virus (22, 73, 76). Contrary to that, another study with a majority African American cohort reveals that dysbiotic gut microbial composition is restored back to uninfected status upon recovery (77). Several confounding factors such as demography, small cohort size, healthy control vs. hospitalized control groups, co-morbidities in the COVID-19 patient group, and antibiotics treatment during hospitalization etc. can contribute to the variation in microbiome observed in different studies. Despite of the variations observed in the composition of dysbiotic microbial communities, the COVID-19 patients' microbiome are consistently depleted in Faecalibacterium (18, 21–23), which has been shown to have anti-inflammatory properties and is recognized as a biomarker of a healthy gut microbiome (20) (Table 1). Gut microbial dysbiosis is also observed in SARS-CoV-2 patients suffering from long-term COVID-19 complications (78). Gut microbial dysbiosis observed in SARS-CoV-2 patients is also accompanied by an altered metabolite profile (20, 79–81). COVID-19 patient fecal samples were found to be enriched in several metabolites, including sucrose, oxalate, 1,5-anhydroglucose, and D-pinitol; the elevated level for some of which remained unchanged at discharge (79). Purine metabolite acids were also depleted in the fecal samples of COVID-19 patients, which can directly impact the repair function of damaged intestinal epithelium (79). On the other hand, the level of metabolites that are known to exhibit immunosuppressive and anti-inflammatory effects such as D-allose and D-arabinose were found to be depleted in patient fecal samples (79). Metagenomic and meta-transcriptomic data reveal significant depletion of short chain fatty acid (SCFA) producing bacterial taxa *Ruminococcaceae* and *Clostridiaceae* (19) and decreased activity of butyrate-producing bacteria, which is reflected in the reduced SCFAs levels observed in fecal samples of COVID-19 patients (73, 82). Depletion of SCFA synthesis pathways was associated with disease severity and showed persistent depletion in patients even after viral clearance (82). Furthermore, a recently published study investigating the role of butyrate releaser (N-(1-carbamoyl-2-phenyl-ethyl) butyramide (FBA) showed promising results in modulating the expression of ACE2, TMPRSS2, and inflammatory cytokines IL-15, MCP-1 and TNF-α in intestinal biopsies (83). SCFAs are also play important role in strengthening intestinal barrier function, regulating inflammation and oxidative stress in intestinal epithelial cells (84). Taken together, regulation of gut microbial homeostasis is not only crucial for the management of clinical symptoms of COVID-19 disease severity but may also be an essential factor for the recovery process after infection.

**Table 1:** Summary of studies exploring SARS-CoV-2 infection associated alteration in the gut microbiome.

**Region/Country**	**Study population**	**Significantly altered bacterial taxa**	**Reference**
Germany	108 COVID-19 patients, 22 Post-COVID-19, 20 Pneumonia control, 26- Asymptomatic controls	↓F. prausnitzii ↓Blautia ↑Parabacteroides ↑Alistipes	(21)
China	15 COVID-19 patients, 6 Pneumonia control, 15 Healthy controls	↓Faecalibacterium prausnitzii ↓Alistipes onderdonkii ↑Clostridium hathewayi ↑Actinomyces viscosus ↑Bacteroides nordii	(23)
China	30 COVID-19 patients, 24 H1N1 patients, 30 Healthy controls	↓*Faecalibacterium*, ↓*Romboutsia*, ↓*Fusicatenibacter*, ↓*Eubacterium hallii*, ↑*Streptococcus*, ↑*Rothia*, ↑*Veillonella*, ↑*Erysipelatoclostridium*, ↑*Actinomyces*	(18)
Italy	15 COVID-19 patients, 8 Hospitalized controls	↑*Proteobacteria*, ↓*Spirochaetes*, ↓*Fusobacteria*	(19)
Hong Kong, China	100 COVID-19 patients, 78 Healthy controls	↓*Bifidobacterium adolescentis*, ↓*Faecalibacterium prausnitzii*, ↓*Eubacterium rectale* ↑*Ruminococcus gnavus*, ↑*Ruminococcus torques*, ↑*Bacteroides dorei*	(22)
China	13 COVID-19 patients, 24 Pneumonia patients, 13 Healthy controls	↓*Bacteroides vulgatus*, ↓*Prevotella copri*, ↓*Clostridium leptum*, ↓*Alistipes putredinis*, ↑*E. coli*, ↑*Akkermansia muciniphila*, ↑*Gemmiger formicili*	(73)

Opioids are also shown to cause significant alteration in gut microbial composition. Abundant evidence from human and animal studies shows the effect of opioids in altering gut microbial composition (85–88). Pre-clinical models showed variation in opioid-induced dysbiotic gut microbiome based on the mode of drug delivery, administration schedule, and animal models. However, opioid use consistently results in increased pathogenic bacteria such as *Enterococcus, Sutterella, Clostridium, Flavobacterium, Fusobacterium*, and decreased commensal microbes, including *Lactobacillus* and *Bifidobacterium* (85, 89). Opioid use has also been shown to increase susceptibility to several pathogenic infections, including *Pseudomonas aeruginosa, Enterococcus faecalis*, and *Listeria monocytogenes*, thereby predisposing toward sepsis and immune dysregulation (90–94). A recent clinical study identified an enrichment of family Bifidobacteriaceae and depletion of Akkermansiaceae among opioid users, along with a significant reduction in fecal SCFA levels (95). Opioid treatment has also been shown to change the level of bacterial metabolites. Animal studies also show that opioid treatment results in significant changes in bacterial metabolites, including bile acids and SCFAs (85, 96). Opioid treatment also causes intestinal barrier disruption and gut bacterial translocation to the peritoneal cavity, liver, and spleen (97, 98).

Gut microbial dysbiosis, altered metabolic profile, and increased inflammatory environment are common denominators in opioid use disorder and SARS-CoV-2 infection. So far, there have been no clinical studies focused on identifying the compounding effect of gut microbial dysbiosis and opioid use on SARS-CoV-2 infection. However, based on available data, it would be reasonable to hypothesize that opioid use may serve as a comorbidity for SARS-CoV-2 infection and can result in poor disease progression and recovery outcomes. Therefore, pre and probiotic therapies to restore microbial homeostasis, and supplementation of metabolites such as SCFA to enhance intestinal barrier integrity may be used to manage SARS-CoV-2 infection and opioid-associated comorbidities on intestinal functions.

## Immuno-Suppressive Role of Opioids

Another life-threatening adverse-effect of opioid use is the increased risk of serious infection due to suppression of the immune response (99). Various *in vitro* as well as *in vivo* experiments have established the immunosuppressive effects of opioids (63). Opioids bind to MOR in the CNS, peripheral neurons, and immune cells, affecting both, the innate and adaptive immune systems (67). Opioids modulate the immune response by a variety of mechanisms which include down-regulating the activity of natural killer-cells, neutrophils, and macrophages, reducing antibody production, dampening the response of B and T cells, and inhibiting cytokine production by macrophages, microglia, and astrocytes (100). When morphine binds to MOR, chemokine receptors on macrophages such as CCR1, CCR2, CXCR1, and CXCR2 are desensitized, phagocytosis is inhibited, and their capacity to kill pathogens is reduced (67). Neutrophil migration is inhibited by opioids, as well their ability to produce superoxide species (67). The effect of opioids on the adaptive immune system includes reduced T-cell apoptosis, impaired T-cell differentiation, modified expression of cytokines, as well as B-cell dysfunction (67).The major histocompatibility complex class II (MHC-II) is compromised by morphine treatment impairing their ability to activate T-cells and switching T-cells toward the T-helper type 2 cells, attenuating the immune response (67). These published data on the immunosuppressive effect of opioids raise concern for the enhanced risk for bacterial and viral infection in severely ill COVID-19 patients admitted to ICU. Morphine treatment has been shown to significantly increase pro-inflammatory cytokines, especially IL-6, IFNγ, and TNFα thereby alleviating hyperinflammatory status (101, 102). Since CRS is one of the major challenges to control the severity of COVID-19 infection, modulation of immune function by morphine and other opioids may have a potential role in treating COVID-19 patients.

A study investigating the effect of opioids on the innate immune response against *Streptococcus pneumoniae* found an 80% increase in mortality after morphine treatment (103). Analysis of lung tissue revealed inflammation in the lungs of the morphine treated mice was amplified (103). This was due to a delay in the recruitment of neutrophils to the lungs at the start of the infection (103). Opioids also dampen the response to viral infection and can enhance replication of viruses such as hepatitis C virus and HIV (104). The interferon system of innate immunity is essential for responding to viral infection promptly and preventing systemic viral dissemination (104). When interferons are secreted, they act as antiviral messengers by triggering cells to express antiviral proteins (104). Type-I IFNs are capable of modulating apoptosis, cell proliferation, and regulating the immune response (104). Opioids suppress the IFN signaling pathway, which causes an increased susceptibility to viral infection (105). A study using single cell RNA sequencing analyzed gene expression for immune subpopulations to investigate the effect of opioids on antiviral genes (105). Compared to control samples, opioid treatment resulted in a suppression of interferon stimulated genes (105). Furthermore, antiviral genes in all immune cell populations were inhibited, affecting both branches of the immune system (106). Overall, opioid use and abuse may lead to more severe lung damage in severely ill COVID-19 patients and significantly increase the chances of bacterial pneumonia.

## Opioid And SARS-CoV-2 Viral Entry

Similar to SARS-CoV, SARS-CoV-2 enters the target cells through the receptor-binding domain (RBD) on viral spike (S) proteins which specifically engages with ACE2 on the target cell surface (107). After receptor binding, host cell-expressed cell surface transmembrane serine protease TMPRSS2 is required for cleavage of S protein and entry of SARS-CoV-2 inside the host cell (108). ACE2 and TMPRSS2 have been detected in the nasal and bronchial epithelium and the highest expression of ACE2 is found in nasal epithelial cells (109). ACE2 and TMPRSS2 co-expression was also found in the esophagus, ileum, and colon which can explain clinically observed viral fecal shedding (71).

**Figure 2:**
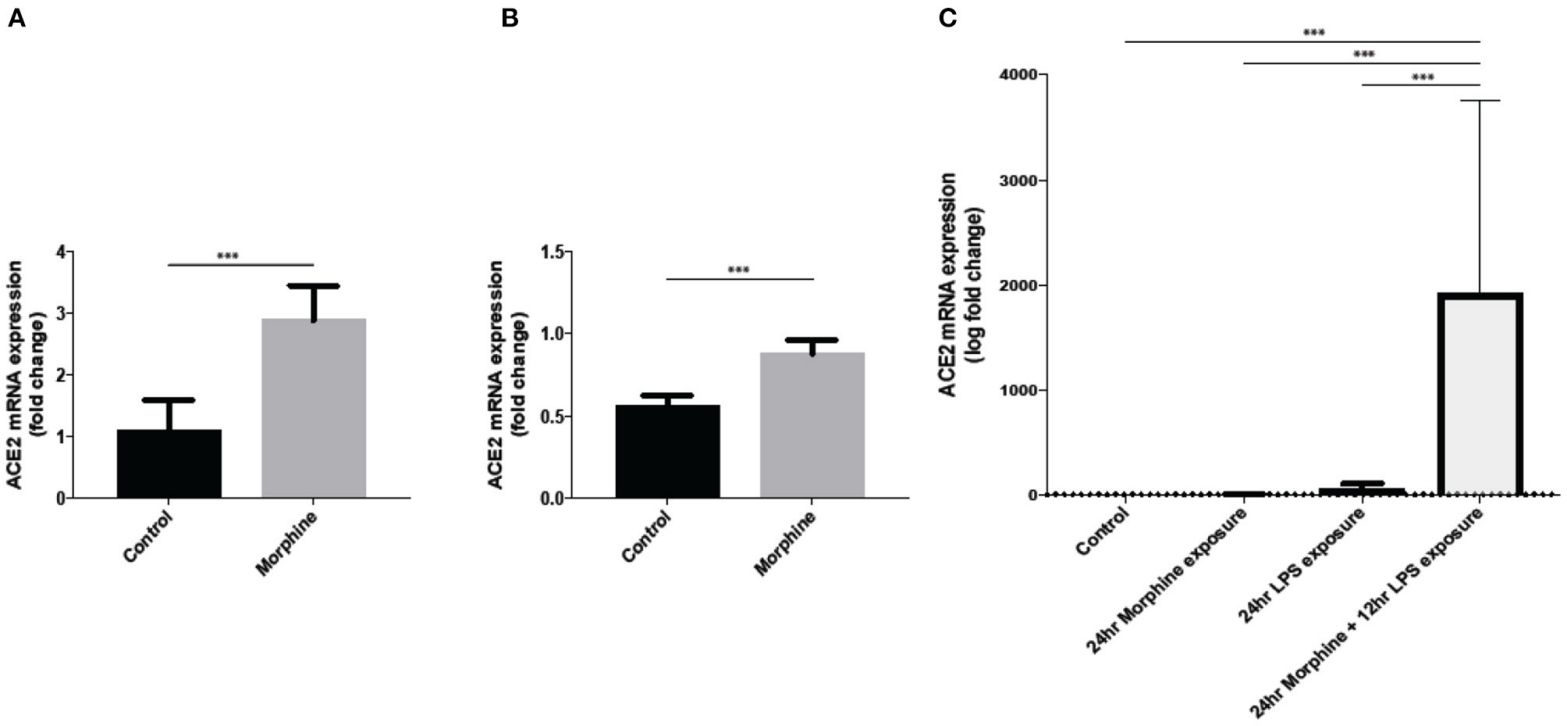
Morphine treatment significantly upregulate ACE2 gene expression in **(A)** mice lung tissue, **(B)** mice brain tissue at 24 h post morphine treatment subcutaneously (*n* = 5), and **(C)** ACE2 expression in SIMA9 cells along with LPS treatment (*n* = 8–10). Data were analyzed by student's *t*-test and one-way ANOVA with *post-hoc* Tukey's test. ***P ≤ 0.001. Mean ± SD.

Limited data is currently available about the impact of opioid use on SARS-CoV-2 infection. In order to study the direct effect of opioids on SARS-CoV-2 viral infection, our unpublished data show that 24-h morphine (25 mg slow-release pellet) treatment in C57BL/6J mice significantly increases the transcription levels of ACE2 in lung tissue (Figure 2A).

Furthermore, our data also show an increased expression of ACE2 in mouse brain tissue after morphine treatment (Figure 2B). Our *in-vitro* data also showed an increased ACE2 expression in microglial cell line SIMA9 after treatment with morphine and LPS (Figure 2C). Since SARS-CoV-2 requires ACE2 to infect host cells, opioid use in COVID-19 patients can further increase the risk for disease severity and also increase the risk of SARS-CoV-2 infection in already vulnerable, severely ill COVID-19 patients and OUD group.

ACE2 expression level can be a significant factor associated with COVID-19 severity (110, 111). Therapies targeting ACE2, such as recombinant human ACE2 (rhACE2) and camostat mesilate are being explored for treating severe COVID-19 (112, 113). Interestingly, according to a recent docking screening conducted by AutoDock Vina, codeine and morphine are found to have a high affinity for ACE2 receptor binding (docking score < −10 Kcal/mol) (114) and can be potentially used as a therapeutic for COVID-19 by blocking ACE2 receptor binding (115). Because of the increased use of prescription opioids in COVID-19 patients and the higher risk of opioid abuse as a result of decreased support, it is essential to focus research on understanding the consequence of opioid use in SARS-CoV-2 infection and replication, disease severity, and recovery.

ACE2 is a component of the renin-angiotensin-aldosterone system which essentially regulates vasoconstriction and blood pressure. Briefly, ACE2 converts angiotensin I (Ang I) to Ang II which causes vasoconstriction, stimulates pro-inflammatory chemokines and promotes inflammation. To counter regulate, ACE2 metabolizes Ang II into Ang-(1-7) and also Ang I into Ang-(1-9) (116). ACE inhibitors (ACEI) and ACE receptor blockers (ARB) have been a part of first line therapy for treating micro and macrovascular complication in hypertension and preventing neuropathy in diabetes patients by upregulating ACE2 expression (117). Since ACE2 is the main receptor for entry of SARS-CoV-2 in cells, the use of ACEIs and ARBs could increase viral entry in patients using these therapies. However, no substantial data has yet been found relating the use of ACEI and ARB and the severity of COVID-19 (118, 119).

In addition to ACE2 and TMPRSS2 proteins, another protein that is a relevant factor in SARS-CoV-2 entry and severity is the aryl hydrocarbon receptor (AhR). AhR is a ligand-activated transcription factor that integrates several cues including environmental, microbial, and metabolic cues to control complex gene transcriptions in a ligand-specific, cell-type-specific, and context-specific manner (120). All cells consistently need sensors such as AhR to adapt and respond to molecular changes in their micromilieu. Upon activation by its ligands, AhR translocate in the nucleus and controls the expression of several target genes that contain AhR-responsive DNA elements in their regulatory regions (120).

Several studies have looked at the importance of the AhR in the mechanisms by which the SARS-CoV-2 virus enters cells of different organs. AhR has been found to regulate the expression of ACE2 by binding to the ACE2 promoter, thus increasing its expression. More specifically, AhR was found to increase ACE2 expression in SARS-CoV-2-infected primates; such increased expression favored a higher viral load and a greater severity of histopathological damage (121). The involvement of AhR in SARS-CoV-2 entry and severity was further established by demonstrating that AhR inhibitors decrease ACE2 expression and viral load and that AhR endogenous agonists stimulate ACE2 gene transcription and protein expression (121). Another study, looking at a link between smoking and susceptibility to coronavirus disease 2019, found cigarette smoke condensates (CSC) to upregulate ACE2 and TMPRSS2 expression in human gingival epithelial cells (GECs) by activating AhR signaling (122). They further demonstrated that AhR depletion decreased SARS-CoV-2 pseudo virus internalization in CSC-treated GECs compared with control GECs. Therefore, these results highlight the importance of studying both exogenous and endogenous ligands activating the AhR receptor and resulting in increased ACE2 expression. Such increased expression reveals itself as important to both the susceptibility to COVID-19 infection and disease severity.

## Melatonin Therapy For SARS-CoV-2 Infection And OUD

Numerous pharmaceutical drugs have been re-examined for their potential efficacy in preventing SARS-CoV-2 infection. Among them, melatonin has brought massive attention to research throughout the COVID-19 pandemic as it has a broad spectrum of antiviral properties, including against three different coronaviruses (123, 124). Moreover, other studies have reported that melatonin is an anti-inflammatory and anti-oxidative molecule with a high safety profile (125). Recent findings demonstrated that melatonin has a potential effect against destructive inflammatory consequences of a SARS-CoV-2 infection by counteracting excessive free radical-mediated oxidative damage and hyper inflammation (126, 127). A recent review by Reiter et al. (128) demonstrated that melatonin counteracts elevated sPLA2-IIA, development of pro-inflammatory M1 macrophages, activation of hypoxia-inducible factor-1α, conversion to Warburg-type metabolism of immune cells, damage to mitochondria, massive release of cytokines, and oxidative stress due to the destructive inflammatory consequences of a SARS-CoV-2 infection (128).

Melatonin treatment also shows a potential role in combating the harmful effects of opiate addiction and opioid-induced behavioral sensitization (129). Using animal models, several studies reported that systemic use of melatonin has an analgesic effect and reverses morphine-induced hyperalgesia and tolerance (130–132). Clinical administration of melatonin is used to treat sleep disorders and mood disorders (133), and therefore can also be helpful in managing poor sleep (insomnia) or disrupted sleep (apnea) commonly associated with OUD (134).

Melatonin is synthesized in the enterochromaffin (EC) cells throughout the gut, and deficiencies in melatonin have been linked to increased permeability of the gut, increasingly associated with a range of diseases (135, 136). A recent study conducted by Zhao et al. demonstrated that melatonin reverses Oxazolone-induced colitis by suppressing type 2 immune response and attenuating intestinal permeability and neutrophil infiltration (137). Melatonin's role in treating intestinal co-morbidities associated with OUD and SARS-CoV-2 infection should be further explored.

## Limitations

The present review has two major limitations. Firstly, most of the studies reviewed here are clinical data gathered from a small cohort of COVID-19 patients from an observational or cross-sectional study, therefore, sometimes presenting contrasting observations based on the age, race, geographic location of the cohort. Secondly, since limited data is availability on the effect of opioid on COVID-19 disease severity and progression, therefore, some information is, in fact, extrapolations about the potential effect of opioids on physiological and neurological manifestation in COVID-19 patients.

## Conclusion

The association between opioid use and risk for infection and developing severe COVID-19 illness should be investigated with better study designs and clinical data analysis. The mechanism underlying the morphine-mediated upregulation of ACE2 in lung tissue and brain is not fully understood and warrants further exploration. Furthermore, a change in the expression profile of ACE2 and TMPRSS2 needs to be evaluated in the nasal epithelium since that is implicated as a portal for initial infection and transmission. Since opioids are also known to cause hospital acquired secondary infections, careful investigation needs to be done to inspect the effect of opioid treatment on secondary infection in severely ill COVID-19 patients requiring mechanical ventilation. Furthermore, the potential role of SCFAs and melatonin should be explored as a therapeutic in SARS-CoV-2 infection and opioid-associated comorbidities.

More research should be done on socio economic conditions that cause OUD thereby increasing infection risk and immune modulations, putting the vulnerable groups at risk of COVID-19 infection. Concentrated efforts should also be done to provide support to OUD patients since they are at high risk for relapse or involuntary withdrawal.

## Author Contributions

RJ and SRo: conceptualization. RJ, DA, and RB: writing-original draft preparation. SRa and SRo: writing-review and editing. RJ, DA, and RD: data acquisition and interpretation. All authors contributed to the article and approved the submitted version.

## Funding

This work was supported by the National Institutes of Health Grants (R01DA044582, R01DA043252, R01DA050542, R01DK117576, and T32DA045734) awarded to SRo.

## Conflict of Interest

The authors declare that the research was conducted in the absence of any commercial or financial relationships that could be construed as a potential conflict of interest.

## Publisher's Note

All claims expressed in this article are solely those of the authors and do not necessarily represent those of their affiliated organizations, or those of the publisher, the editors and the reviewers. Any product that may be evaluated in this article, or claim that may be made by its manufacturer, is not guaranteed or endorsed by the publisher.
